# Deletion of the BDNF Truncated Receptor TrkB.T1 Delays Disease Onset in a Mouse Model of Amyotrophic Lateral Sclerosis

**DOI:** 10.1371/journal.pone.0039946

**Published:** 2012-06-27

**Authors:** Sudhirkumar U. Yanpallewar, Colleen A. Barrick, Hannah Buckley, Jodi Becker, Lino Tessarollo

**Affiliations:** Neural Development Section, Mouse Cancer Genetics Program, Center for Cancer Research, National Cancer Institute, Frederick, Maryland, United States of America; University of Dayton, United States of America

## Abstract

Brain Derived Neurotrophic Factor (BDNF) exerts strong pro-survival effects on developing and injured motoneurons. However, in clinical trials, BDNF has failed to benefit patients with amyotrophic lateral sclerosis (ALS). To date, the cause of this failure remains unclear. Motoneurons express the TrkB kinase receptor but also high levels of the truncated TrkB.T1 receptor isoform. Thus, we investigated whether the presence of this receptor may affect the response of diseased motoneurons to endogenous BDNF. We deleted TrkB.T1 in the hSOD1^G93A^ ALS mouse model and evaluated the impact of this mutation on motoneuron death, muscle weakness and disease progression. We found that TrkB.T1 deletion significantly slowed the onset of motor neuron degeneration. Moreover, it delayed the development of muscle weakness by 33 days. Although the life span of the animals was not affected we observed an overall improvement in the neurological score at the late stage of the disease. To investigate the effectiveness of strategies aimed at bypassing the TrkB.T1 limit to BDNF signaling we treated SOD1 mutant mice with the adenosine A2A receptor agonist CGS21680, which can activate motoneuron TrkB receptor signaling independent of neurotrophins. We found that CGS21680 treatment slowed the onset of motor neuron degeneration and muscle weakness similarly to TrkB.T1 removal. Together, our data provide evidence that endogenous TrkB.T1 limits motoneuron responsiveness to BDNF in vivo and suggest that new strategies such as Trk receptor transactivation may be used for therapeutic intervention in ALS or other neurodegenerative disorders.

## Introduction

Neurotrophins are potent regulators of survival and function of mammalian nervous system neurons [1], [2], [3]. These functions are mediated by their binding to the Trk tyrosine kinase receptors and p75, a member of the tumor necrosis factor receptor family. Trk genes include three members, TrkA which binds the neurotrophin Nerve Growth Factor (NGF), TrkB the main receptor for BDNF and Neurotrophin4/5 (NT4/5), and TrkC the preferred receptor for Neurotrophin3 (NT3) [4], [5], [6]. The TrkB and TrkC loci, in addition to the full-length kinase receptors, can generate, by alternative splicing, truncated receptors lacking the canonical intracellular tyrosine kinase domain [reviewed in [6]]. However, how these receptor isoforms coordinate cellular or systemic neurotrophin responses is still poorly understood. Gene targeting experiments in mouse deleting all, or a specific isoform, of the TrkB or TrkC gene have shown that the full-length Trk kinase receptors have the strongest prosurvival effect on specific populations of the nervous system [7], [8], [9], [10]. Truncated receptors appear to affect neuronal viability only when over-expressed in artificial or pathological situations [11], [12], [13]. Nevertheless, they are the most highly expressed isoforms in the adult mammalian brain [14], [15], [16]. Only recently it has became apparent that physiological truncated Trk receptors can have multiple functions in mammalian development. For example, TrkB.T1 deletion in mouse causes increased anxiety and morphological abnormalities in basolateral amygdala neurons consistent with an independent signaling function for this receptor. Moreover, physiological levels of TrkB.T1 receptors are important regulators of full-length TrkB (TrkB.FL) signaling in vivo since loss of TrkB.T1 can partially rescue BDNF haploinsufficiency [8]. One issue that has not yet been addressed is whether physiological levels of truncated TrkB receptors can affect specific pathologies. For example, it has been reported that the frontal cortex of patients with Alzheimer's disease has decreased BDNF and TrkB.FL expression accompanied by increased truncated TrkB expression [17]. Moreover, in ALS patients, BDNF mRNA and protein are dramatically upregulated in muscle and total TrkB mRNA is increased in the spinal cord. Yet, phosphorylation of the TrkB receptor is reduced suggesting that TrkB signaling impairments in ALS are not caused by insufficient neurotrophin supply but rather by a mechanism affecting the TrkB response to BDNF [18], [19].

Although TrkB or BDNF are not required for motoneuron survival during development, BDNF/TrkB kinase signaling is required for maintenance of the cholinergic phenotype of adult motor neurons and prevents embryonic and postnatal motoneuron cell death in a variety of experimental paradigms [reviewed in [20]]. These observations have led to the clinical evaluation of BDNF as a therapeutic agent in patients affected by amyotrophic lateral sclerosis (ALS), a neurodegenerative disorder affecting motor neurons in the spinal cord, brainstem and motor cortex [21]. However, to date BDNF has failed to benefit ALS patients in clinical trials [22]. Moreover, most recently it has also been suggested that specific deletion of TrkB.FL from motoneurons in an ALS mouse model can be beneficial through a still unknown mechanism [23].

Here we investigated whether the TrkB.T1 receptor isoform affects motoneuron survival and disease progression in a widely used ALS mouse model that carries a transgene with a human familiar Gly^93^→ALA mutation in the copper/zinc superoxide dismutase gene (SOD1^G93A^ or SOD1 mice) [24], [25]. Our results suggest that the deletion of TrkB.T1 prevents the loss of motoneurons and muscle function in SOD1 mice at the early disease stage without affecting the survival of the animals. Moreover, treatment with the adenosine A2A receptor agonist CGS21680, that has been shown to enhance survival of lesioned facial motoneurons [26] leads to a similar delay in the onset of the disease [27]. Since, CGS21680-mediated activation of TrkB occurs independently of BDNF and it is not influenced by the presence of TrkB.T1 at the membrane, our data suggest that TrkB.T1 expression may affect the ability of diseased motoneurons to respond to endogenous BDNF.

## Results

### TrkB.T1 deletion delays motoneuron loss in SOD1 mutant mice

**Figure 1:**
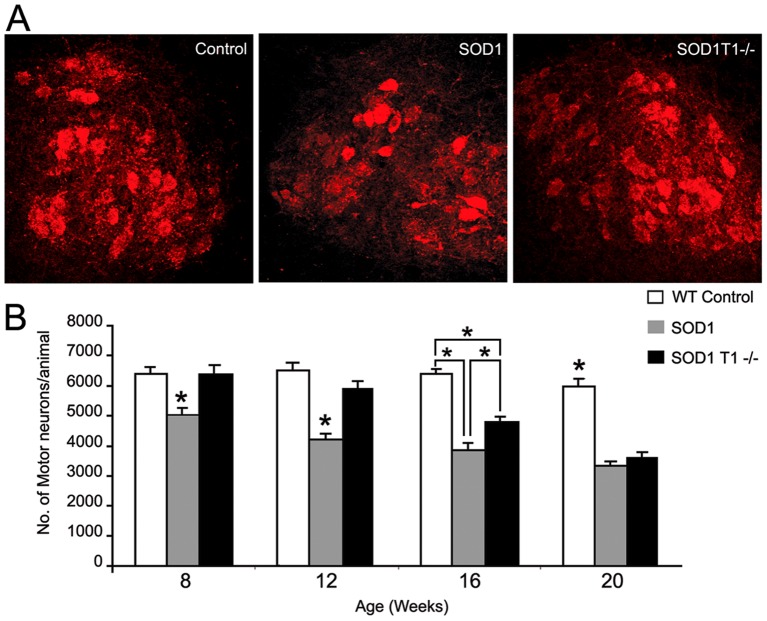
Effect of TrkB.T1 deletion on motoneuron degeneration. (A) Representative immunofluorescent images of lumbar spinal cord showing ChAT-positive motoneurons in 8-week-old animals. In comparison to age-matched WT controls, SOD1 transgenic mice show a small but significant loss of motoneurons. This loss was prevented by deletion of TrkB.T1 in these mice (SOD1T1−/−). (B) Histogram showing number of motor neurons at 8, 12, 16 and 20 weeks. Cell counts show a progressive decrease in number of motoneurons in SOD1 animals as compared to WT controls. The deletion of TrkB.T1 in SOD1 transgenic mice completely rescues this loss at 8 and 12 weeks. At 16 weeks this neuroprotection is partial and significant whereas at 20 weeks it is completely lost such that both the SOD1 and SOD1T1−/− groups show severe reduction in motoneuron numbers as compared to WT animals. The data are the Mean ± SEM. * indicates P<0.05. N = 6 in each group. Statistical analysis by ANOVA followed by post-hoc Tukey test.

Since motoneurons express high levels of TrkB.T1 we decided to investigate whether this receptor can limit motoneuron responsiveness to endogenous BDNF and affect their survival in pathological conditions. For this we employed the G93A^SOD1^ mutant mouse, a well-studied mouse model of ALS. Crosses between TrkB.T1^−/−^ (T1−/−) and SOD1^G93A^ (SOD1) mutant mice were such to obtain control (WT), SOD1 and SOD1;T1−/− mutant mice at 8, 12, 16 and 20 week (Wk) of age in order to evaluate the effect of TrkB.T1 deletion on motoneuron numbers in the SOD1 mice (Fig. 1). At 8 weeks, the SOD1 animals already showed a small (∼20%) but significant decrease in the number of choline acetyltransferase positive (ChAT+) motor neurons, which reached a 35% loss by 12 Wk and 40% by 16 Wk as compared to control animals. Interestingly, SOD1;T1−/− mice start to show a marginal, non significant loss of neurons only at 12 weeks. However, by 16 Wk, the deletion of TrkB.T1 provided only a partial protection since SOD1;T1−/− mice had ∼15% more motoneurons than SOD1 animals. At 20 weeks, deletion of TrkB.T1 did not confer any protection to SOD1 mutant motoneurons since both SOD1;T1−/− and SOD1 animals lost about 45% motoneurons compared to WT mice (Fig. 1). Although the loss of the cholinergic phenotype in spinal cord motoneurons has been associated with motoneuron cell death [24], [28], [29], to rule out the possibility that deletion of TrkB.T1 may affect ChAT expression but not cell survival, we counted motoneurons from Cresyl Violet stained sections at 12 wk of age. Again, we found that only SOD1 mutant mice had lost a significant number of motoneurons (∼35%; p<0.05) while SOD1;T1−/− mutant mice had a small non significant (∼8%) loss of neurons (7156.66±423.37 in WT, 4588.33±440.33 in SOD1; 6550±345.14, in SOD1;T1−/−). Taken together these data suggest that TrkB.T1 delays the loss of SOD1 mutant motoneurons and does not affect their cholinergic phenotype.

### TrkB.T1 deletion affects muscle strength in SOD1 mutant mice at disease onset

**Figure 2:**
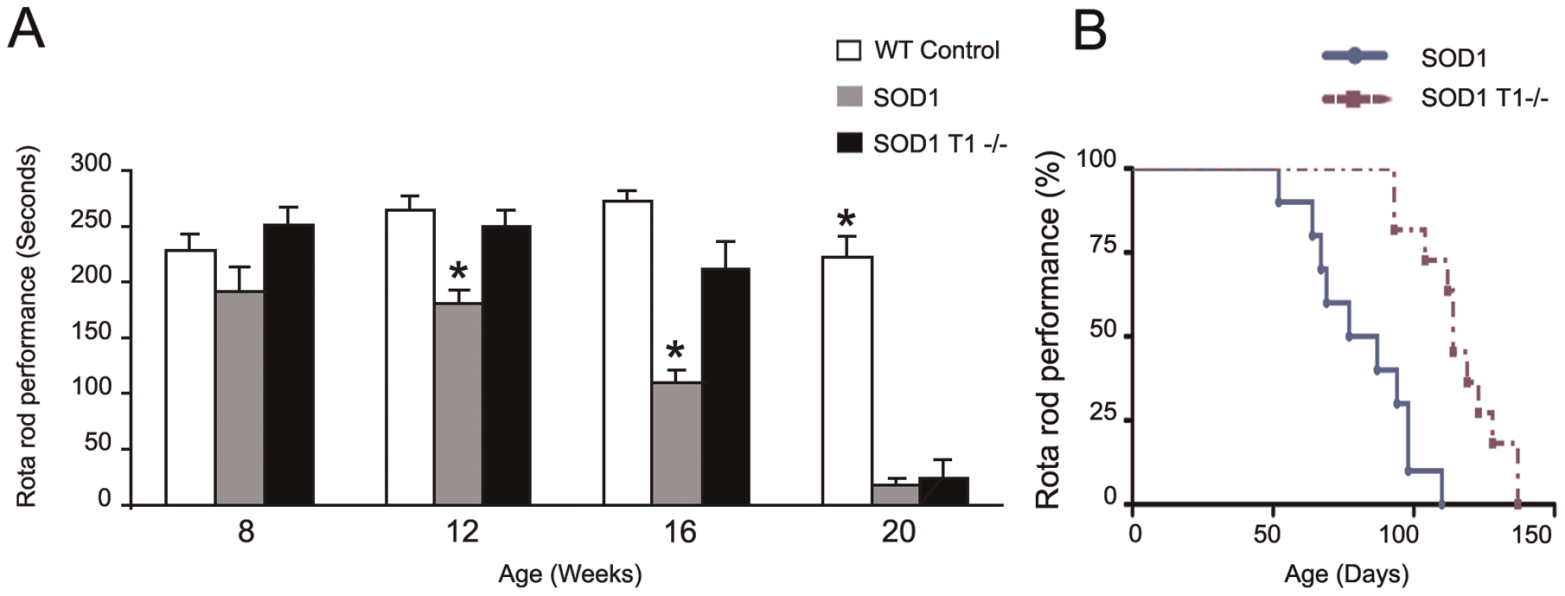
Deletion of TrkB.T1 improves the motor performance of SOD1 transgenic mice on rotarod in the early phase of the disease. (A) Histogram showing the rotarod performance of WT, SOD1 and SOD1T1−/− animals at 8, 12, 16 and 20 weeks. At 8 weeks, both SOD1 and SOD1 T1−/− animals perform similarly to controls although SOD1 mice show some impairments. With disease progression, the SOD1 transgenic mice show a significant reduction in the amount of time spent on the rotarod at 12 and 16 weeks as compared to controls. A slight loss of motor performance is evident only at 16 weeks in the SOD1T1 −/− animals, although by 20 weeks the SOD1 and SOD1;T1−/− groups are indistinguishable. The data are the Mean ± SEM. * P<0.05. N≥7. Statistical analysis by ANOVA followed by post-hoc Tukey test. (B) Kaplan-Meier analysis of the SOD1 and SOD1T1−/− mice rotarod performance in relation to their age. Note that deletion of TrkB.T1 in the SOD1 transgenic mice delays the impairment in rotarod performance by 34 days (81.6±5.87 days, n = 10 in SOD1 versus 115.8±6.80, n = 7 in SOD1 T1−/−, p<0.05).

To determine whether deletion of TrkB.T1 improves muscle strength of 8 to 20 Wk old SOD1 mice, we tested their ability to stay on an accelerating rotarod over 5 min (Fig. 2A). At 8 weeks, we did not observe any significant difference between groups (Fig. 2). At 12 weeks the performance of the SOD1 mutant animals significantly decreased by 22% compared to controls while the SOD1;T1−/− mouse performance began to decline (23%) by 16 weeks. However, as observed in the motoneuron number analysis (Fig. 1), we found that by 20 weeks SOD1 and SOD1;T1−/− mutant mice were indistinguishable in their performance (Fig. 2A). In addition, we also compared the rate of decline in rota-rod performance over age using Kaplan-Meier statistics as indicated in Fig. 2B. Overall, the comparison of performance curves between SOD1 and SOD1;T1−/− mutant mice shows that the impairment occurs 33 days later in SOD1;T1−/− mutant mice (Fig. 2B) suggesting that the motoneurons rescued by TrkB.T1 deletion in the early stage of the disease are functional.

### Disease progression in SOD1;TrkB.T1−/− mutant mice

**Figure 3:**
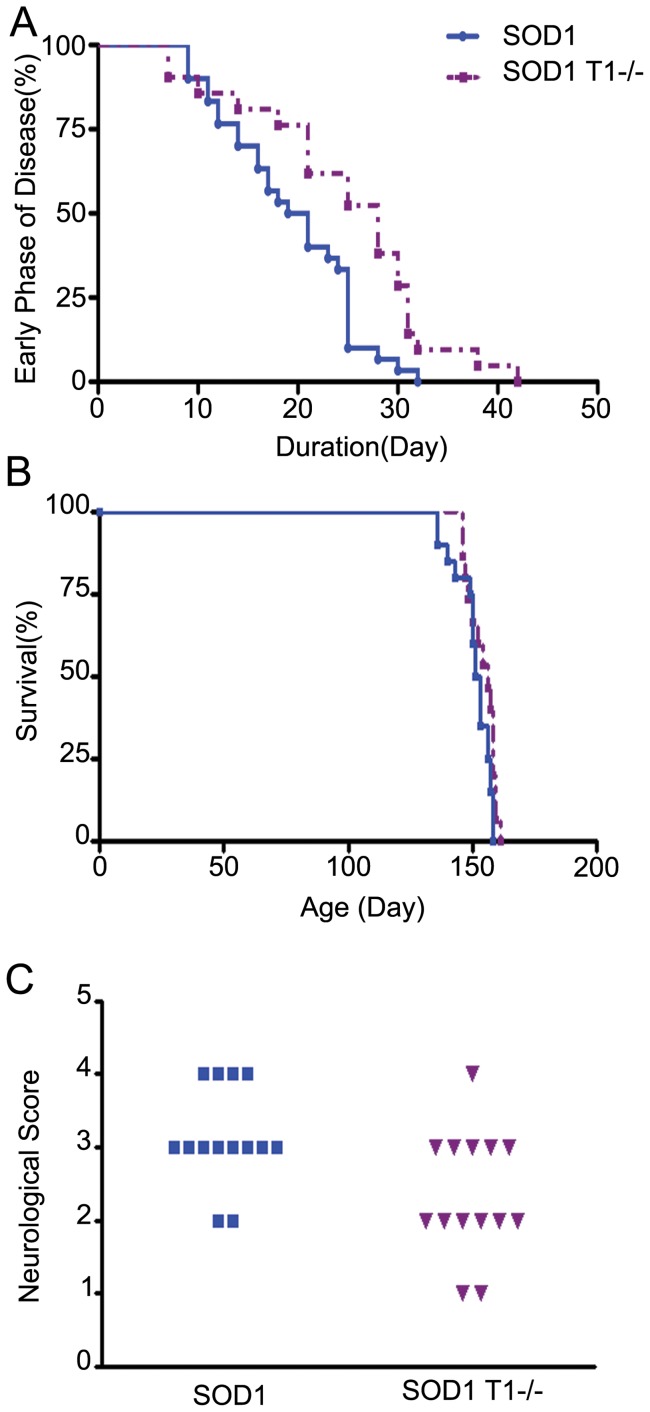
Disease progression and survival of SOD1 and SOD1;T1−/− mice. Histograms from Kaplan-Meier analysis showing the duration of the early phase (A) and the end stage (B) of the disease, as well as the neurological scoring (C) of SOD1 and SOD1;T1−/− mice. Deletion of TrkB.T1 delays the early phase of disease progression by 6 days (19.2±1.25 days, n = 30 in SOD1 animals versus 25.2±2.09 days n = 21 in SOD1;T1−/−, P<0.05; A). Note the similar life span between groups (B) despite the improvement in the overall neurological score at 20 wk in the SOD1;T1−/− compared to the SOD1 mutant mice (C; P<0.05).

To determine whether the improvement in motor neuron survival and muscle weakness observed after deletion of TrkB.T1 affects disease progression, we studied a cohort of SOD1 and SOD1;T1−/− mice over their lifespan for signs and symptoms of disease. Decline in 10% of their body weight is used as a reference point for the early phase of the disease [30]. We found that TrkB.T1 deletion extended the mean duration of the early phase by 6 days in SOD1 mutant mice (a 31% increase; Fig. 3A). However, we did not observe any difference in the mean lifespan of the two groups (Fig. 3B). In addition to comparing the survival curves, we evaluated the disease signs and symptoms in 20-Wk-old animals by analyzing their locomotor activity using established neurological scores [Fig. 3C; [28]]. Although, at this age we found that both motoneuron numbers and rota-rod performance are indistinguishable between SOD1 and SOD1;T1−/− mice, SOD1 transgenic mice predominantly scored in the 3–4 range, a reflection of severe impairments in locomotor activity due to muscle weakness and paralysis, while deletion of TrkB.T1 in these animals improved the score to the 2–3 neurological range. These data suggest a partial but significant improvement in muscle function in mice at the end stage of the disease (n = 14 in each group, P<0.05; Fig. 3C).

### The adenosine A2A receptor agonist CGS21680 (CGS) delays disease onset of SOD1 mutant mice

**Figure 4:**
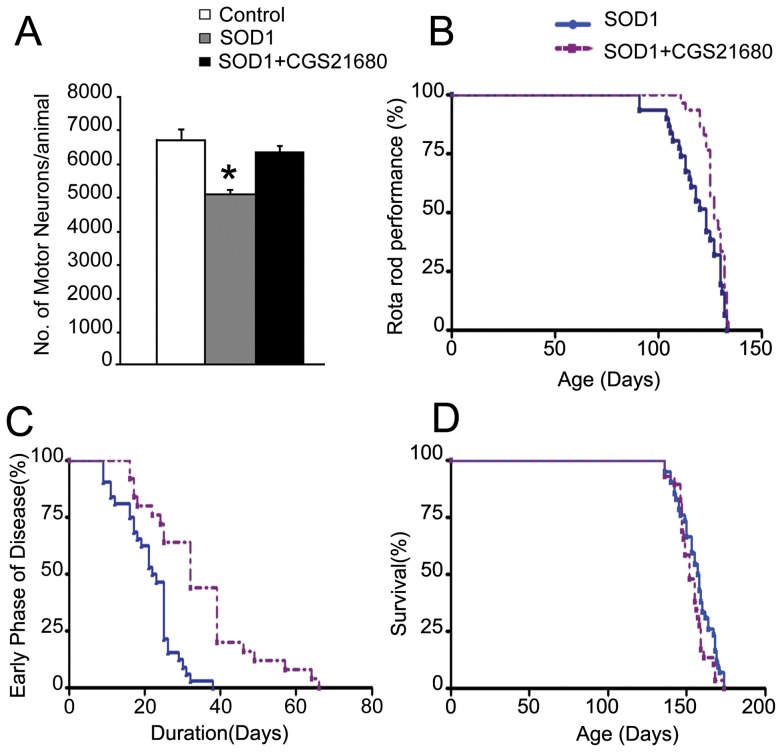
The adenosine A2A receptor agonist CGS21680 delays disease onset in SOD1 mutant mice. CGS treatment at 12 weeks of age significantly rescues motor neuron degeneration in SOD1 mice (A; p<0.05). Analysis of rotarod data showing that treatment of SOD1 mice with CGS delays the impairment in rotarod performance by 7 days compared to untreated SOD1 mice (B; Kaplan Meier Analysis, p<0.01). CGS treatment also prolongs the duration of the early phase of the disease by 12 days (C; p<0.05) although it does not affect the mean life span of the SOD1 mutant animals (D).

The delayed disease onset caused by deletion of TrkB.T1 in the SOD1 mutant mice suggests that the presence of this receptor may affect pro-survival effects of TrkB kinase on diseased motoneurons by limiting their responsiveness to BDNF. To further test this hypothesis we investigated whether we could pharmacologically affect TrkB signaling in motoneurons by using the adenosine agonist CGS that has been shown to phosphorylate TrkB.FL in motoneurons irrespective of the presence of BDNF and therefore also TrkB.T1 [26], [27]. SOD1 transgenic mice received daily injections of CGS starting at 8 weeks of age before the clinical manifestation of the disease. To evaluate whether CGS treatment could prevent motoneuron loss in SOD1 mice, we sacrificed a group of mice at 12 weeks and evaluated the number of surviving motor neurons. While Cresyl Violet staining of spinal cords revealed a significant decrease in the number of motoneurons in the SOD1 mice compared to controls, SOD1 mutant mice treated for 4 wks with CGS had motoneuron numbers similar to those of SOD1;T1−/− mutant and WT mice (Fig. 4A). Furthermore, analysis of muscle strength by rota-rod analysis showed a delay in the development of rota-rod impairment in the CGS treated SOD1 mice by 8 days when compared to the vehicle-treated SOD1 controls (Figure 4B) suggesting that CGS could affect both survival and function of SOD1 motoneurons. Moreover, CGS treatment also prolongs the early disease stage by 12 days (Figure 4C). However, as seen in the SOD1;T1−/− mutant mice, protection of motoneurons by CGS disappeared in the late stages of the disease as CGS treated SOD1 mutant mice had no increased life span compared to that of controls (Figure 4D).

## Discussion

In this study, we tested the hypothesis that the presence of endogenous truncated TrkB limits BDNF pro-survival effects on diseased motoneurons. We found that deletion of TrkB.T1 receptors from the SOD1^G93A^ mouse model of ALS delays disease onset without extending the lifespan of the animals. Moreover, we showed that the adenosine A2A receptor agonist CGS, that causes TrkB phosphorylation independently of BDNF by a transactivation mechanism, has an effect similar to removing TrkB.T1 in this ALS mouse model.

The pro-survival role of BDNF on lesioned facial and sciatic nerves as well as its protective effect, in combination with CNTF, on degenerating motoneurons in the wobbler mutant model has lead to its use as a potential therapeutic agent in neurodegenerative motoneuron diseases [31]. However, in clinical trials BDNF has failed to show any beneficial effect [reviewed in [22]]. Although this failure has been attributed to factors such as inadequate dosage, short half-life and limited bioavailability, even a subsequent trial with intrathecal infusion of BDNF, aimed at improving the bioavailability of BDNF, also failed. Thus, the precise reasons for the lack of efficacy of BDNF in the clinic are still elusive. Curiously, the activity and/or appropriate delivery of BDNF has never been evaluated in animal models of ALS and, although insufficient neurotrophic support has been included as one possible reason for motoneuron degeneration, the levels of BDNF and its receptor TrkB are not decreased in ALS. In fact, some studies have shown that BDNF and total TrkB levels are either increased or unaltered in the CNS of ALS patients. Despite this, some studies have reported a decrease in TrkB phosphorylation [18], [19], [32], [33]. These observations suggest that while a primary deficiency of BDNF may not be a cause for the reduction in TrkB activity, alterations of the level of TrkB receptor isoforms or in downstream signaling events could underlie motoneuron losses. Our data support this hypothesis since removing physiological TrkB.T1 receptors delays the onset of motoneuron losses and functional impairments. Therefore, developmental up-regulation of TrkB.T1 in the adult CNS, not only influences TrkB signaling in normal physiological conditions but also the response of injured motoneurons to BDNF. Recently, it has been reported that TrkB.T1 up-regulation occurs under conditions of neuronal insults such as excitotoxicity and cerebral ischemia [34], [35]. Moreover, TrkB.T1 is up-regulated in neurodegenerative disorders such as Alzheimer's disease [17] suggesting that imbalances in expression between the TrkB.FL and truncated TrkB isoforms makes BDNF signaling ineffective and contributes to neuronal damage [35]. In accordance with this hypothesis, over-expression of TrkB.T1 induces motor neuron death and in the CNS worsens the infarct area after cerebral ischemia [11], [36]. However, whether physiological levels of TrkB.T1 can influence neuronal survival during injury has not been tested. Our data now support the idea that endogenous TrkB.T1 negatively affects neuron survival in neurodegenerative disorders.

Moreover, our data suggest that delivery of neurotrophins may not be sufficient to promote neuronal survival of injured neurons because TrkB.T1 limits neurons responsiveness to BDNF. Interestingly, axotomized retina ganglionic cells survive indefinitely when BDNF is supplied in combination with TrkB overexpression, but survive only for a short period if provided with BDNF alone [37].

While in vitro experiments and artificial overexpression experiments have limitations, our study strongly suggests that physiological or pathological imbalances in TrkB receptor isoform levels can affect motoneuron survival. It will be of interest to investigate whether ALS patients with increased total TrkB, and decreased TrkB phosphorylation have imbalances in TrkB isoform expression levels. The recent identification of ALS patients with mutations in the TDP-43 and FUS genes, that appear to be involved in splicing regulation suggest that this could be a possibility if, for example, TDP-43 and/or FUS can affect TrkB splicing [38]. Interestingly, in the TS16 trisomic mouse model the triplication of a number of genes, including some controlling splicing, causes an upregulation of TrkB.T1 receptors responsible for increased cortical neuronal cell death and hippocampal neuron vulnerability to BDNF deficiency [12]. New studies aimed at identifying whether ALS patients have higher levels of truncated TrkB receptors and whether mutations in the TDP-43 and FUS genes affect TrkB splicing should help clarify this issue.

Despite the decrease in motor neuron losses and amelioration in muscle function at the early stage of the disease, deletion of TrkB.T1 failed to increase the mean life span of the SOD1 mutant animals. ALS is a complex neurodegenerative disease for which there is little information about the molecular mechanism causing motoneurons to degenerate [39]. Even the pathogenic effect of the SOD1 mutant protein on motoneurons is still unclear. However, it has been shown that there is a direct correlation between the level of SOD1 mutant protein and the severity of the phenotype suggesting a toxic effect of the mutated protein [39], [40]. Contrary to the SOD1 familial cases, which carry only one allele of the mutant gene, the ALS mouse model used in the present study carries a high transgene copy number and shows an extremely rapid progression of disease. Therefore, the extreme toxicity of the mutant SOD1 protein produced at such a high level might hide small but significant effects on survival that could be detected if less SOD1 mutant protein was produced. The present study aimed to increase the endogenous BDNF-TrkB.FL signaling by deleting the putative dominant/negative function of TrkB.T1 without providing exogenous neurotrophin support. In such a scenario, it is likely that deletion of TrkB.T1, which provides neuroprotection in the early stages of the disease is unable to sustain its effects with the increasing load of SOD1 mutant protein that occurs at later stages.

Alternatively, the increased trophic effect could be limited to motoneurons of the limbs and not of respiratory muscles, which would lead to an overall improvement of the clinical manifestation of the disease in both the early and late stages of the disease (Fig. 3C) but to a failure in the overall extension of the animal lifespan. A differential effect on specific motoneuron populations could be explained by different levels of expression of TrkB.T1 in distinct motoneuron pools. For example, TrkB.T1 deletion would have a more significant impact on BDNF signaling in a neuronal population normally expressing high levels of the receptor compared to the response of a neuronal population expressing lower levels of TrkB.T1. However, to date it is still unknown whether different motoneuron pools express TrkB.T1 at different levels.

The report that TrkB.T1 affects Ca++ signaling suggests that imbalances in the TrkB.T1 receptor level may affect motoneuron survival by altering intracellular Ca++ levels [12], [41]. Dysregulation of calcium homeostasis in spinal motoneurons of SOD1 mutant mice has already been reported [42], [43]. Moreover, it has also been shown that spinal motor neurons have diminished calcium-buffering capacity due to their low expression of calcium binding proteins, making them more vulnerable in disease states like ALS [44]. Thus, it is conceivable that deletion of TrkB.T1 might improve calcium homeostasis in SOD1 mutant mice contributing to the rescue of spinal motoneuron death in the early stages of disease. In this scenario, the delay in the onset of the disease in SOD1 mutant mice caused by deletion of TrkB.T1 and CGS treatment could be due to different mechanisms: one based on Ca++ level regulation by TrkB.T1 and the other based on the increased activation of TrkB.FL signaling by transactivation.

One of the most surprising findings of this study was the positive effect, at least during the early stage of the disease, caused by TrkB.T1 removal on motoneuron survival without the need to apply exogenous BDNF. Thus, these data support the relevance of physiological levels of TrkB.T1 receptors on the control of TrkB signaling promoting the survival of diseased motoneurons. While our genetic strategy (i.e deletion of TrkB.T1) provides the proof-of-principle that targeting the pathways downstream of BDNF can be one important factor in achieving motoneuron neuroprotection, our results also suggest that it may be more useful to promote activation of TrkB intracellularly rather than by using BDNF mimetics. In fact, the use of the adenosine A2a receptor agonist CGS achieved the same results as deleting TrkB.T1. Of course, it is also possible that besides transactivating TrkB, activation of the adenosine receptor could have an effect by promoting cAMP signaling, that, in turn, induces the release of neurotrophic factors including BDNF, NGF and GDNF [45]. If this is the case, treatment of SOD1 mutant mice with CGS, coupled with the TrkB.T1 deletion may have additive effects and address this issue.

Treatment of SOD1^G93A^ mutant mice with CGS delays motoneurons death similarly to TrkB.T1 deletion suggesting that the pro-survival effect seen in motoneurons by removing TrkB.T1 is cell autonomous. In fact, CGS causes activation of TrkB.FL that is expressed exclusively in motoneurons and TrkB.T1 removal may improve TrkB.FL activation bypassing TrkB.T1 dominant/negative or neurotrophin-sequestering activity. However, since TrkB.T1 is expressed in both motoneurons as well as glia cells, in the future it will be critical to investigate the effect of deletion of this receptor isoform in specific cell types to establish the cell-specificity of TrkB.T1 rescue. Indeed recent data from a mouse model of experimental autoimmune encephalomyelitis (EAE) have shown that deletion of TrkB from glia cells renders these mice more resistant to EAE susceptibility and severity. Mechanistically, TrkB.T1 in astrocytes appears responsible for the production of nitric oxide which in turns induces neuronal degeneration and apoptosis [46].

Despite the repeated therapeutic failure of neurotrophic factors in clinical trials, recent experimental and clinical efforts are aimed at improving the level of neurotrophic factors in motoneurons by gene therapy and/or by developing novel systems to deliver drugs [47]. Additional efforts are also focusing on the effects of the combinatorial use of different neurotrophic factors. Irrespective, our data suggests that a better understanding of the signal transduction pathways downstream of neurotrophins and of modalities to activate these pathways may be more critical than identifying strategies for delivering exogenous neurotrophins.

## Materials and Methods

### Animals

SOD1^G93A^ [B6SJL-TGN(SOD1-G93A)1GUR/J; 002726] mice were acquired from The Jackson Laboratory (Bar Harbor, ME) and crossed to TrkB.T1 knockout mice backcrossed on C57/B16J background for at least 10 generations [12]. All experiments were conducted with the experimenter blind to the genotype of the animals. Protocols followed the National Institutes of Health Guidelines for animal care and use, and were approved by the NCI-Frederick ACUC committee.

### Motor neuron survival and stereology

Mice were anesthetized and perfused transcardially with 0.9% NaCl followed by 4% paraformaldehyde. The lumbar region of the spinal cord (L1–L5) was removed and 50-µm transverse sections were cut on a cryostat. Every 10^th^ section (17–20 sections total per animal) was collected for analysis. Motoneurons were visualized by immunofluorescent staining for Choline acetyltransferase (ChAT, 1∶500 Millipore) and by Cresyl Violet staining and counted by the stereological modified optical fractionator method multiplying the number of counted neurons per section with the sampling fraction.

### Evaluation of motor performance

Muscle strength and coordination were assessed by an accelerating rota-rod (Ugo Basile, Italy) that rotates from 4 to 40 RPM over 5 min [29]. After two training trials of 5 min each (at 7 weeks) animals were run three times a week (from 8 weeks on) and the duration for which the animal stayed on the rota-rod without falling (up to a maximum of 300 seconds) was recorded. We also determined the age at which the performance of the animals fell below 150 seconds in two successive trials, and this time point was taken as an index of rota-rod impairment.

### Analysis of disease progression and survival

To monitor the progression of the disease we used two different parameters: early progression of disease and end-stage disease in the animals. Starting at 8 weeks of age, animals were weighed three times weekly. Given that animals start losing weight with disease progression, we defined the early progression of disease as the duration between the time of peak weight and the time when animals lose 10% of peak weight due to muscle atrophy. End-stage was defined as the age of actual death of the animal or the age at which paralysis rendered the animal unable to right itself within 20 seconds when placed on its side [30].

### Neurological Scoring

Animals were scored for disease signs and symptoms at 20 weeks based on the following scale [28]:

0 =  normal functions, no sign of disease onset.

1 =  distal muscle (paw) weakness of one limb.

2 =  distal muscle weakness of two limbs, slight wobble when walking.

3 =  weakness of proximal muscle (leg) of one limb plus weakness in two distal muscles, clear wobble when walking.

4 =  both hind limbs paralyzed with dragging of hind limbs.

5 =  animal is immobile.

### CGS21680 treatment

The adenosine A2A receptor agonist CGS21680 was purchased from Tocris (USA) or custom synthesized from ChemieTek (IN, USA). CGS21680 was dissolved in 5% DMSO prior to use. Starting at 8 weeks of age animals received a single daily intraperitoneal injection of CGS21680 or vehicle (5% DMSO) and were sacrificed at different time points depending on the experiments. The dose of CGS21680 (5mg/kg) was determined on the basis of our pilot study and other studies [27], [48].

### Statistical Analysis

GraphPad software (InStat and Prism) was used for all statistical analysis. Comparisons of motor neuron numbers and rota-rod performance between the groups at different ages was done by ANOVA followed by post-hoc Tukey-Kramer Multiple comparison test or Dunn test. Survival analysis (rota-rod impairment, early progression of disease and end-stage) was subjected to Kaplan-Meier Statistics. A Log rank P value was calculated to compare the survival curves. Neurological scores were compared by Mann-Whitney test. Data are represented as Mean ± SEM.
